# Case Report: “Dumbbell” giant right coronary artery ectasia with right atrial fistula

**DOI:** 10.3389/fcvm.2025.1498359

**Published:** 2025-02-28

**Authors:** Jianggui Shan, Heng Wang, Song Xue

**Affiliations:** Department of Cardiovascular Surgery, Shanghai Jiao Tong University School of Medicine Affiliated Renji Hospital, Shanghai, China

**Keywords:** cardiovascular surgery, case report, computed tomography angiography, coronary artery ectasia, coronary artery fistula

## Abstract

A 50-year-old female patient presented with a “dumbbell” giant right coronary artery ectasia, characterized by two artery dilation segments both reaching the level of a giant aneurysm with a normal segment between them. Computed tomography angiography showed a fistula sac in the right atrium. The vessel shape was a typical type IV (localized or segmental) coronary artery ectasia, which is rarely seen on true imaging. The patient had a 3-year history of chest tightness, without dyspnea, worsened by physical activity. Additional tests indicated that she had mitral valve regurgitation, superficial myocardial bridge, and anemia, all of which led to the development of her symptoms. She felt relieved after successful coronary artery fistula repair, mitral valvuloplasty, and fistula sac removal. At the 6-month follow-up, no complications were found according to echocardiography. Patients with coronary aneurysms can be asymptomatic in the early stage, while this case indicates that the dumbbell shape may be a developing stage of giant coronary aneurysm whose origin and close-fistula segments are influenced by separate hydrodynamics during ectasia or aneurysm formation.

## 1 Introduction

Coronary artery fistula has an estimated prevalence of 0.002% in the general population, and fistula drainage sites can be found in the aorta, pulmonary artery, and all four heart chambers (1). Coronary artery ectasia, defined as diffuse vessel dilation >1.5 times that of the adjacent normal segment, is a specific coronary aneurysmal dilation with a prevalence in the range of 1.2%–4.9% (2). Some patients can be asymptomatic with coronary artery ectasia and/or fistula, while surgical intervention is necessary and can be effective in cases with cardiovascular comorbidities to prevent further complications.

Here, our team reports a rare case of type IV segmental ectasia with dilating segments located at both ends of the right coronary artery (RCA), from the origin to the fistula opening, which lacks sufficient real images (3). Both dilating segments met the criteria for giant coronary artery aneurysm [internal diameter (ID) >20 mm or >4× ref. ID] (3).

## 2 Case presentation

### 2.1 Patient information and timeline

A 50-year-old woman had a 3-year history of chest tightness without dyspnea. Her symptoms worsened after physical activity. She denied any past medical history of hypertension or diabetes. No significant family or psychosocial history was reported. She was menopausal on arrival at the hospital.

The patient reported having an echocardiogram 3 years before at a local institution, which indicated an RCA fistula to the right atrium (RA) and mitral regurgitation; however, no therapy was provided as she was unwilling to undergo a thoracotomy at that time. As her symptoms worsened, she had a second echocardiogram with similar results at another local institution 1 month before this admission, and she decided to receive treatment. She was initially admitted to the cardiology department of our institution and then transferred to our department 3 days later.

### 2.2 Clinical findings and diagnostic assessment

The patient’s vital signs upon admission were as follows: axillary temperature 36.4℃; heart rate 76 beats/min; respiratory rate 17 breaths/min; and blood pressure 98/43 mmHg. Transthoracic echocardiography (TTE) showed a left ventricular ejection fraction (LVEF) of 67% (ref. ≥54%). Laboratory tests showed a brain natriuretic peptide (BNP) level of 111.0 pg/mL (ref. 0.0–100.0 pg/mL), a platelet count of 397 × 10^9^/L (ref. 125–350 × 10^9^/L), and a lipoprotein (a) level of 892 mg/L (ref. 0.0–300.0 mg/L).

**Figure 1:**
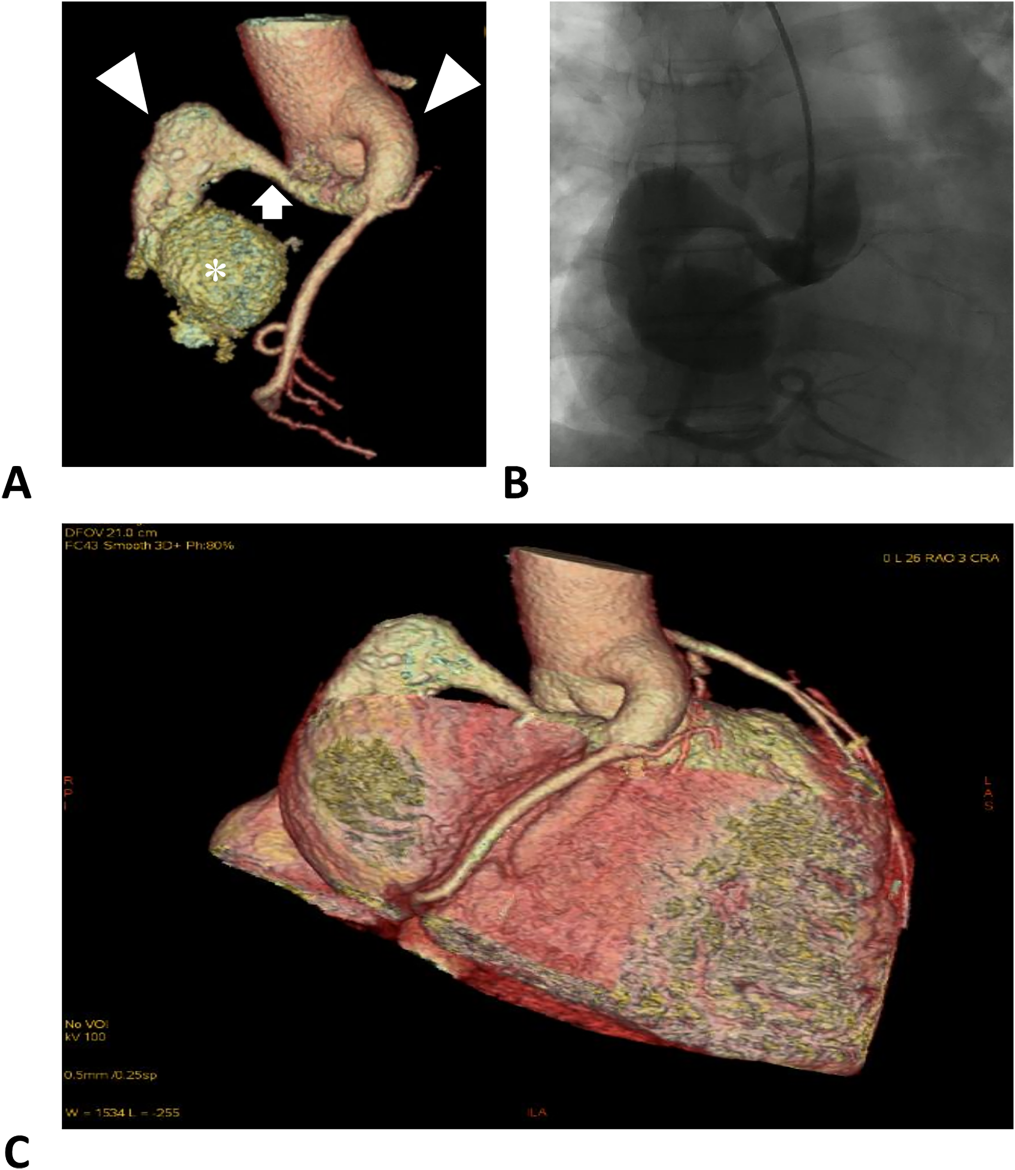
Right coronary artery images. **(A)** Computed tomographic angiography of the RCA. The left and right triangles denote the distal and proximal aneurysmal segments of the RCA. The arrow denotes a normal segment between the dilations, forming a dumbbell-shaped ectasia. The asterisk denotes the fistula sac formed in the right atrium. **(B)** Digital subtraction angiography of the RCA. **(C)** Reconstruction of the coronary arteries and the heart, illustrating the positional relationship.

**Figure 2:**
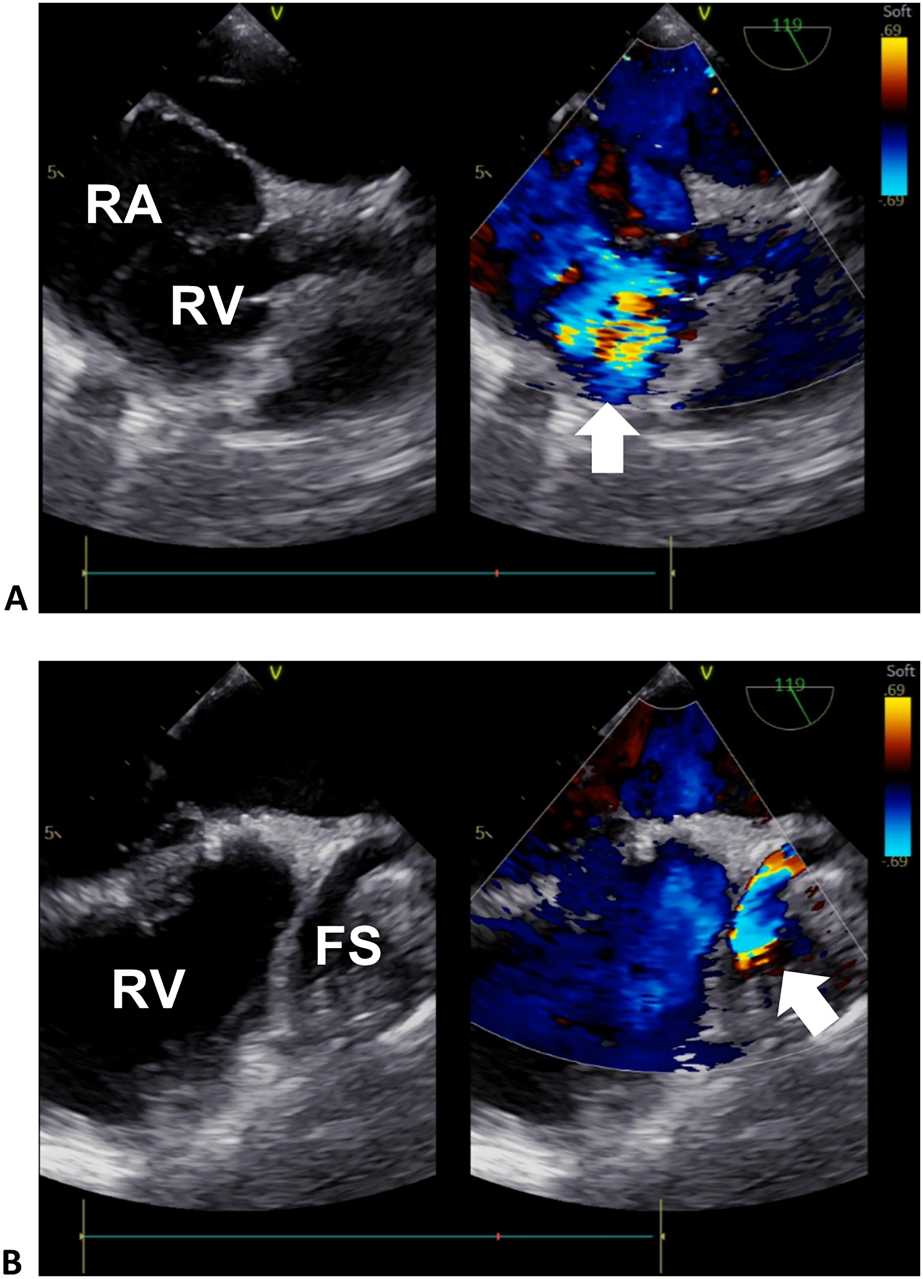
Transesophageal echocardiogram of the right heart. **(A)** Sectional plane showing the exit flow from the fistula sac into the right heart. **(B)** Sectional plane showing the entrance flow from the RCA to the fistula sac. The arrows denote the turbulence signals of the exit and entrance flows in **(A)** and **(B)**, respectively. RA, right atrium; RV, right ventricle; FS, fistula sac.

Computed tomography angiography and digital subtraction angiography showed a “dumbbell” RCA ectasia at the origin (ID 14 mm) and the fistula wall in the RA (ID 37mm × 36 mm, forming a fistula sac), and the connecting segment was normal (Figure 1, Supplementary Material Video S1). The ostium from the RCA fistula sac to the RA was 10 mm in width, facing the inferior vena cava. Transesophageal echocardiogram (TEE) showed a continuous flow enhanced during diastole at the entrance (peak velocity 2.5 m/s) and exit (peak velocity 1.8 m/s) of the RCA fistula sac (Figure 2, Supplementary Material Videos S2, S3). Her atria (left ID 44 mm, ref. 27–38 mm; right vertical diameter 62 mm, ref. <53 mm), left ventricle (end of diastole ID 61 mm, ref. 39–52 mm), and pulmonary artery (ID 32 mm, ref. 12–26 mm) were enlarged.

Additional laboratory and auxiliary tests indicated the following: (1) anemia (hemoglobin 71 g/L), with lowered mean corpuscular volume (MCV) of 66.5 fl (ref. 82–100 fl), mean corpuscular hemoglobin (MCH) of 17.4 pg (ref. 27–34 pg), mean corpuscular hemoglobin concentration (MCHC) of 264 g/L (ref. 316–354 g/L), and lowered ferritin of 5.4 μg/L (ref. 11–306.8 μg/L), serum iron of 2.5 μmol/L (ref. 7.8–32.2 μmol/L); (2) moderate mitral regurgitation (with an enlarged mitral annulus of 38 mm, ref. 21–32 mm); and (3) superficial myocardial bridge in the left anterior descending branch [50% compression during systole, thrombolysis in myocardial infarction (TIMI) flow grade III]. These cardiovascular comorbidities may have complicated effects on symptom development. No evidence of coronary atherosclerosis, infective endocarditis, or pulmonary disease was found.

### 2.3 Therapeutic intervention and follow-up

The patient received a dual antiplatelet therapy (100 mg aspirin, 75 mg clopidogrel, q.d., PO), atorvastatin calcium tablets (20 mg/tablet, q.n., PO), rabeprazole sodium enteric-coated capsules (10 mg/capsule, q.d., PO), potassium chloride sustained release tablets (0.5 g/tablet × 2, b.i.d., PO), ferrous succinate tablets (0.4 g/tablet, q.d., PO) for the first 3 days. After being transferred to our department, the patient received recombinant human erythropoietin (10^4 ^IU, q.o.d., SC); ferrous succinate tablets (0.2 g/tablet, q.d., PO) were also given for anemia. The hemoglobin (76 g/L), MCV (64.7 fl), MCH (17.2 pg), and MCHC (266 g/L) were not much improved 1 day before surgery.

**Figure 3:**
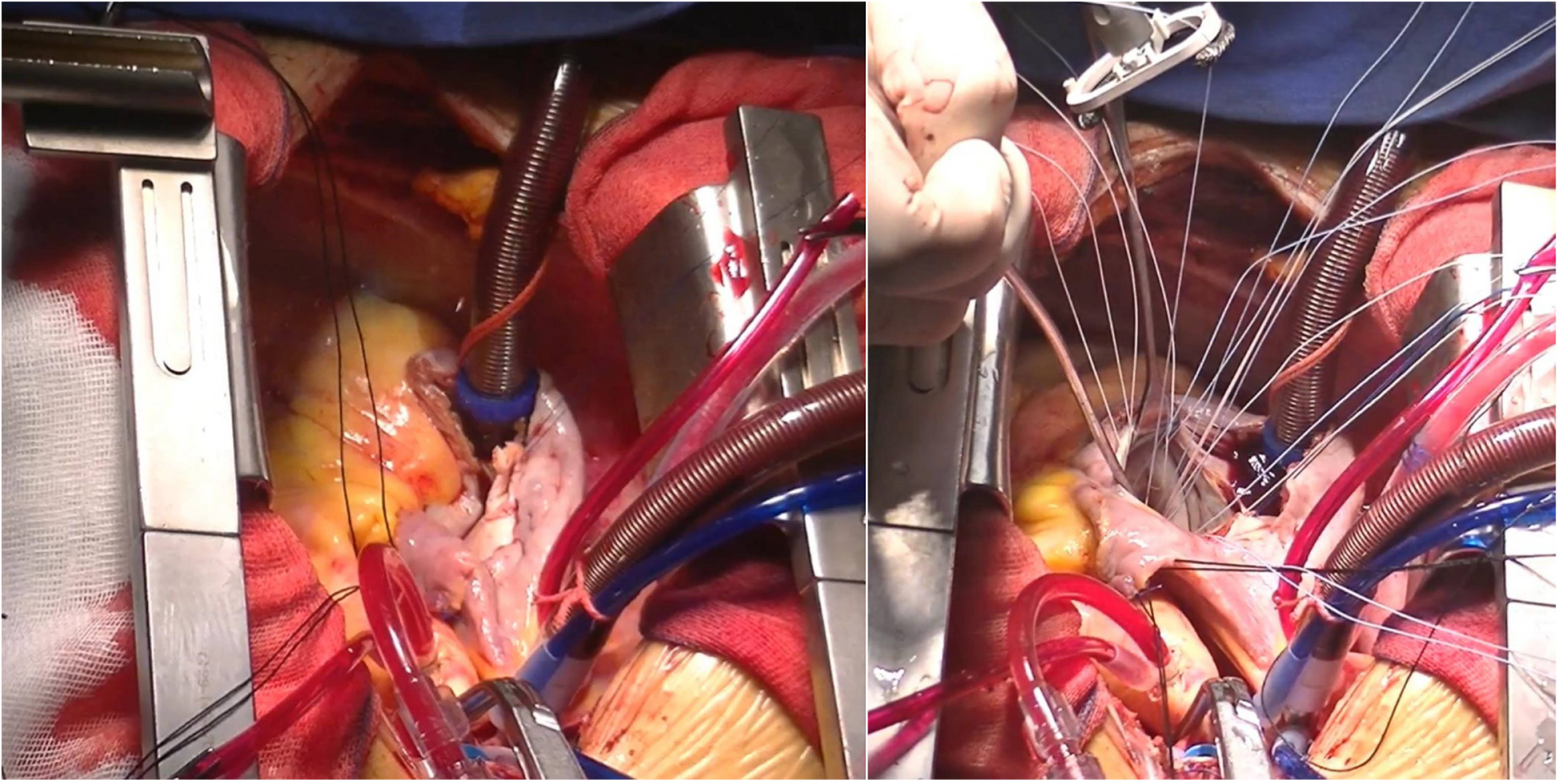
Surgical procedures. Left panel: right coronary artery to right atrial fistula repair. Right panel: mitral valvuloplasty.

Five days after transfer, the patient underwent cardiopulmonary bypass-assisted coronary artery fistula repair and mitral valvuloplasty (Figure 3), and the RA fistula sac was removed. Her postoperative TEE showed no RA fistula flow (Supplementary Videos S4, S5). The patient experienced symptom relief postoperatively. Since anemia may increase the risk of postoperative cardiovascular events, acute kidney injury, and mortality, indices including D-dimer, creatinine, and BNP were monitored (4). Her anemia did not significantly worsen postoperatively (hemoglobin 71 g/L). After a 10-day hospital stay, she was discharged with prescriptions for warfarin sodium tablets (2.5 mg/tablet, q.d., PO), digoxin tablets (0.13 mg, q.d., PO), metoprolol tartrate tablets (12.5 mg, b.i.d., PO), spironolactone tablets (20 mg/tablet, b.i.d., PO), potassium chloride sustained release tablets (0.5 g/tablet × 2, b.i.d., PO), and hydrochlorothiazide tablets (25 mg/tablet × 2, q.d., PO).

Six months after discharge, the patient experienced no cardiovascular symptoms. She self-reported having no complications or other health events. The TTE found no regurgitation or other complications at the mitral valve, and the LVEF was 69%. The fistula was well repaired with no leakage, and the ID of the RCA origin remained at 15 mm. The patient chose not to have additional invasive examinations.

## 3 Discussion

The RCA is involved in 50%–60% of coronary artery fistulae, and 19%–26% of fistula drainage sites are located in the RA (1). The RCA-RA fistula can lead to aneurysm formation. Of patients with coronary artery fistula, 5.9% experience a coronary artery aneurysm (5). A computer simulation of hydrodynamics in an RCA-right ventricle fistula model found (1) high fluid velocity and large wall shear stress, and (2) high pressure at the distal and proximal RCA segments, respectively, and both abnormalities can be corrected by fistula closure (6). Except for this specific case, the initial anatomy and local vulnerability of coronary arteries may affect dilation development, forming different aneurysm shapes (7). The dumbbell-shaped dilation of the RCA found in our patient may have been influenced by its unique hydrodynamics. Other adult congenital cases of RCA fistula presented similar RCA deformation (Supplementary Table S1). Interestingly, RCA origin or proximal segment dilation and distal giant aneurysm can exist separately or simultaneously, indicating that they may be influenced by independent fluid forces. It is reasonable to assume that, without correction, the hydrodynamic aberration could have continued to accelerate the progression of dilation.

A number of patients with coronary artery fistula can be asymptomatic, especially in the early stages of giant aneurysm formation. However, several factors can lead to significant symptoms or signs. First, the turbulent flow can result in local thrombogenesis, potentially leading to acute coronary syndrome (8, 9). Second, the enlarged RCA can exert an occupying effect that compresses adjacent tissues. Third, blood diversion can cause myocardial ischemia, functional valve regurgitation, infective endocarditis, and late heart chamber enlargement, which increases the risk of heart failure and arrhythmia (10). Dyspnea and chest pain are the most common symptoms reported in RCA fistula cases, while palpitations, fever, and other infectious symptoms can also be seen in these patients (Supplementary Table S1). Although functional tricuspid or pulmonary valve regurgitation is more common in RCA-RA fistula for the left-to-right diversion, our case had an uncommon mitral valve regurgitation instead. Similar to a previous report of a patient with an early-stage RCA-RA fistula and relatively normal morphology but mitral insufficiency who experienced chest pain, the mitral valve regurgitation in our patient may have contributed to her chest tightness (11). In addition, the myocardial bridge can add to myocardial ischemia caused by blood diversion, which exacerbates the symptom during exertion. Our team concluded that the patient’s symptoms resulted from the combined effects of multiple heart conditions.

In conclusion, the dumbbell shape may represent an early stage in the development of a larger giant RCA aneurysm, with the RCA origin and close-fistula segments being influenced by separate hydrodynamic forces during ectasia formation.

## Data Availability

The original contributions presented in the study are included in the article/Supplementary Material, further inquiries can be directed to the corresponding author.
