# Supplementary material for: Case Report: “Dumbbell” giant right coronary artery ectasia with right atrial fistula
Source: Front Cardiovasc Med. 2025 Feb 28;12:1498359. doi: 10.3389/fcvm.2025.1498359 (PMC11906408; doi:10.3389/fcvm.2025.1498359)
Supplement: Supplementary file 1 [file Table1.pdf]

Supplementary Table.1 Similar right coronary artery fistula cases.*

Case	Age	Sex	chief complaint or first diagnosis	Right coronary artery dilation	Comorbidity	Ref
1	71	Male	Coronary artery atherosclerosis	Giant aneurysm (6.5×5.4×5.4) (cm)	hypertension, hypercholesterolaemia, peripheral vascular disease	(1)
2	40	Female	Stable angina, continuous heart murmur since junior high	Dilated segment (13.2mm) at the origin	None	(2)
3	73	Female	Malaise, fever, reduced appetite, upper abdominal discomfort, lethargy (due to infective endocarditis), continuous cardiac murmur at first admission; atrial fibrillation, functional tricuspid regurgitation, lowered ejection fraction during the 3-year follow-up.	Ectasia	None	(3)
4	72	Female	Palpitations, supraventricular tachycardia, dizziness and light headedness	Giant aneurysm (3.4×3.3×3.0) (cm) in the serpiginous tract	Osteoporosis, patent foramen ovale	(4)
5	60	Male	Acute worsening of renal function and erythroderma (due to infection)	Aneurysm at the distal segment close to the fistula	None	(5)
6	46	Male	Dyspnea and chest distress	Ectasia	None	(6)
7	64	Female	Chest tightness, shortness of breath	Giant aneurysm at distal segment (8.5×7.5×7.0) (cm)	Hypertension, atrial fibrillation	(7)
8	49	Male	Dyspnea and chest pain	Ectasia	hypertension and hypercholesterolaemia	(8)
9	50	Female	dyspnea, fever, weight loss, lower back discomfort, tachycardia (due to infective	Ectasia	None	(9)

			endocarditis)			
10	59	Female	Coronary artery fistula (asymptomatic)	Aneurysm (2.5cm) at the distal segment close to the fistula	None	(10)
11	42	Female	Angina and dyspnea	Giant aneurysm (8.0×6.0) (cm)	None	(11)
12	54	Female	Chest pain	Ectasia	Rheumatoid arthritis, lung fibrosis	(12)
13	70	Female	Continuous heart murmur	Ectasia (16mm) and aneurysm at the distal segment	None	(13)
14	74	Female	Dyspnea and chest pain	Ectasia (20-40mm)	None	(14)
15	25	Female	Angina	Dilation (21mm) at the proximal segment	None	(15)
16	55	Male	Coronary artery aneurysm (asymptomatic)	Giant aneurysm at distal segment (6.5×4.8) (cm)	None	(16)
17	57	Female	Chest pain	Giant aneurysm	None	(17)
18	62	Male	Palpitations, dyspnea	Ectasia (up to 30mm)	None	(18)
19	67	Female	Palpitations, chest pain	Dilation (5-12mm) at the origin and proximal segment	None	(19)
20	46	Male	Chest tightness and pain	Not found	Coronary artery atherosclerosis	(20)
21	67	Male	Orthopnoea	Dilation at the proximal segment and giant aneurysm (6.0×5.0) (cm) in the atrial septal	None	(21)
22	67	Male	Heart failure	Aneurysm in the right atrioventricular groove	None	(22)
23	30	Female	Palpitations, chest pain, shortness of	Dilation (9mm) at the proximal	Migraines	(23)

			breath	segment		
24	45	Female	Fatigue with intermittent dry cough and dyspnea, heart murmur	Dilation (20mm) at origin, aneurysms in the RCA and interatrial septum	None	(24)
25	29	Female	Continuous cardiac murmur	Ectasia	None	(25)
26	42	Male	Dyspnea and chest pain	Ectasia	None	(26)
27	43	Female	Dyspnea	Dilation (15-30mm) at the origin and proximal segment	None	(27)
28	74	Male	Hypertension	Giant aneurysm (10×5×6) (cm)	None	(28)
29	60	Male	Paroxysmal atrial fibrillation	Ectasia	Hypertension and hyperuricaemia	(29)
30	49	Male	Cardiomegaly	Enlarged coronary sinus	None	(30)
31	63	Male	Chest pain, palpitations,	Giant aneurysm	Secundum atrial septal defect,	(31)
32	20	Male	Fever, headache, dyspnea	Dilation at the origin (5.2mm), dilated sinonodal artery (2.1×1.7) (cm)	None	(32)
33	70	Female	Lower extremity edema, dyspnea	Ectasia (10mm), giant aneurysm (3cm) at the distal segment	None	(33)
34	63	Male	Angina	Ectasia	paroxysmal atrial fibrillation, mild asthma, Addison's disease, recurrent urinary tract infections	(34)
35	50	Female	Continuous heart murmur	Ectasia	None	(35)
36	69	Female	Dyspnea, pedal edema, jugular venous distention, bibasilar rales, hepatomegaly, ascites, severe pitting edema	Ectasia	None	(36)

37	36	Female	Palpitations, short of breath, chest pain	Giant aneurysm (6.0×4.0) (cm)	None	[37]
38	66	Male	Dyspnea	Giant aneurysm	Hypertension, dyslipidemia, coronary artery atherosclerosis	[38]
39	29	Male	Fever, headache (due to infective endocarditis)	Dilation (26.9mm) at the origin	None	[39]
40	61	Female	Right atrial myxoma	Ectasia	None	[40]
41	26	Male	Cough, shortness of breath, palpitations	Ectasia	None	[41]
42	38	Female	Dyspnea, short of breath	Dilation at the proximal segment	None	[42]
43	36	Male	Exercise intolerance, recurrent dizziness, palpitations	Ectasia	None	[43]
44	53	Female	Fever, headache, vomiting	Ectasia	None	[44]
45	31	Male	Enlarged cardiac silhouette	Ectasia	None	[45]

*. Including cases of right coronary artery (cameral) fistulas whose drainage sites are the right atrium and coronary sinus.

REFERENCES

1. Omair M, Roubos N, Johns J, Srivastava P. Giant right coronary artery aneurysm with fistula into the right atrium, *BMJ Case Rep*. (2019) 12:e231666. doi: 10.1136/bcr-2019-231666
2. Yokoyama S, Nagao K, Higashida A, Aoki M, Yamashita S, Yamashita A, et al. Surgical repair of a right coronary aneurysm with a coronary artery fistula to the right atrium, *J Surg Case Rep*. (2021) 2021:rjab286. doi: 10.1093/jscr/rjab286
3. How WJ, Luckie M, Bratis K, Hasan R, Malik N. Evolving consequences of right coronary artery to right atrium: coronary cameral fistula-a case report, *Eur Heart J Case Rep*. (2024) 8:ytae207. doi: 10.1093/ehjcr/ytae207
4. Acker AM, Ibrahim ME, Acker MA. Surgical management of an aneurysmal coronary cameral fistula to the right atrium, *Glob Cardiol Sci Pract*. (2021) 2021:e202128. doi: 10.21542/gcsp.2021.28
5. Takahashi Y, Sasaki Y, Shibata T, Bito Y, Suehiro S. Successful surgical treatment of a mycotic right coronary artery aneurysm complicated by a fistula to the right atrium, *Jpn J Thorac Cardiovasc Surg*. (2005) 53:661-4. doi: 10.1007/BF02665081
6. Pu L, Li R, Yang Y, Liu G, Wang Y. Right coronary artery coronary sinus fistula with coronary sinus ostium stenosis, *Echocardiography*. (2017) 34:1102-4. doi: 10.1111/echo.13559
7. Wang C, Zhu W, Chen M, Zheng Y, Fan X. Giant right coronary artery aneurysm with right coronary artery-right atrial fistula, *Coron Artery Dis*. (2024) 35:78-9. doi: 10.1097/MCA.0000000000001307
8. Barr J, Acharya MN, Kourliouros A, Raja SG. Technical Considerations of Giant Right Coronary Artery Aneurysm Exclusion, *Case Rep Surg*. (2016) 2016:3795640. doi: 10.1155/2016/3795640

9. Green T, Crilley J. Endocarditis and coronary artery fistula: a case report, *Eur Heart J Case Rep*. (2018) 2:yty023. doi: 10.1093/ehjcr/yty023
10. Lee S, Song S, Cho HS. Isolation and bypass approach for Treating a large coronary artery aneurysm with Concomitant fistula to the right atrium: A case report, *Asian J Surg*. (2024) :S1015-9584(24)01962-6 [pii]. doi: 10.1016/j.asjsur.2024.08.222
11. Ahmad T, Pasarad AK, Kishore KS, Maheshwarappa NN. Huge aneurysm and coronary-cameral fistula from right coronary branch: First case, *Asian Cardiovasc Thorac Ann*. (2016) 24:181-6. doi: 10.1177/0218492314553819
12. Bittencourt MS, Seltman M, Achenbach S, Rost C, Ropers D. Right coronary artery fistula to the coronary sinus and right atrium associated with giant right coronary enlargement detected by transthoracic echocardiography, *Eur J Echocardiogr*. (2011) 12:E22. doi: 10.1093/ejechocard/jeq180
13. Araki R, Abe H, Adachi H, Umekawa S, Hattori S, Noda Y, et al. A case of coronary artery fistula diagnosed in the right decubitus position by transthoracic echocardiography, *J Clin Ultrasound*. (2011) 39:493-6. doi: 10.1002/jcu.20805
14. Burgazli KM, Atmaca N, Mericliler M, Cetin SM, Evagelopoulos N, Erdogan A, et al. Giant aneurysm of the right coronary artery with fistula to right atrium, *Eur Rev Med Pharmacol Sci*. (2013) 17:2457-9
15. Erdogan E, Cap M, Topel C, Efe SC, Gurbuz AS. Coronary artery fistula detected with transesophageal echocardiography: An unexpected cause of pulmonary hypertension and chest pain, *Echocardiography*. (2018) 35:2127-9. doi: 10.1111/echo.14162
16. Ren Y, Xie L, Ruan W, Li Y, Ji P, Gan C, et al. Fistula occlusion and ligation for a giant right coronary artery aneurysm concurrent with right atrial fistula: a case report, *BMC Surg*. (2019) 19:166. doi: 10.1186/s12893-019-0624-3
17. Kodaira M, Itoh T, Koizumi K, Numasawa Y. Giant right coronary artery aneurysm complicated by a fistula to the right atrium, mimicking a ruptured sinus of Valsalva aneurysm, *BMJ Case Rep*. (2018) 2018:bcr2018226197. doi: 10.1136/bcr-2018-226197
18. Filizcan U, Ugurlucan M, Zencirci E, Caglar IM, Filik ME, Cetemen S, et al. Right coronary artery aneurysm with right atrial fistula, *J Card Surg*. (2011) 26:381-2. doi: 10.1111/j.1540-8191.2011.01251.x
19. Yilmaz R, Demirbag R, Gur M. Echocardiographic diagnosis of a right coronary artery-coronary sinus fistula, *Int J Cardiovasc Imaging*. (2005) 21:649-54. doi: 10.1007/s10554-005-3858-3
20. Liu J, Yu Z, Wang G. Coronary Artery Fistula and Severe Coronary Artery Stenosis: A Case Report and an Insight for Potential Pathogenesis of Coronary Artery Atherosclerosis, *Int Med Case Rep J*. (2024) 17:227-33. doi: 10.2147/IMCRJ.S442878
21. Osada H, Kanemitsu N, Meshii K, Ohnaka M. Giant atrial septal aneurysm originating from the right coronary artery, *Interact Cardiovasc Thorac Surg*. (2016) 23:329-31. doi: 10.1093/icvts/ivw099
22. Fukuda K, Handa S, Ogawa S, Ohnishi S, Nakamura Y. Noninvasive evaluation of right coronary artery-right atrial fistula using two-dimensional echocardiography, pulsed Doppler echocardiography and color flow mapping, *Cardiology*. (1988) 75:375-80. doi: 10.1159/000174402
23. Mirza S, Nanda NC, Baweja G, Misra V, Pacifico A. Multiple fistulae connecting the right coronary artery to the coronary sinus, *Echocardiography*. (2004) 21:199-202. doi: 10.1111/j.0742-2822.2004.03072.x
24. Marullo AG, Sabik JF. Right coronary artery and interatrial septal aneurysms with fistulous connection to the right atrium, *Ann Thorac Surg*. (2002) 73:969-70. doi: 10.1016/s0003-4975(01)03186-1
25. Mandegar MH, Saidi B, Roshanali F. Right coronary artery fistula to the right atrium, *Pediatr Cardiol*. (2011) 32:1053-4. doi: 10.1007/s00246-011-0016-7
26. Schmid FX, Elsner D, Merk J, Birnbaum DE. Giant right coronary artery-coronary sinus fistula, *Eur J*

Cardiothorac Surg. (2002) 21:929. doi: 10.1016/s1010-7940(02)00080-5
27. Shrivastava V, Akowuah E, Cooper GJ. Coronary artery aneurysm with a fistulous connection to the right atrium mimicking a sinus of Valsalva aneurysm, *Heart*. (2003) 89:e4. doi: 10.1136/heart.89.1.e4
28. Abou Eid G, Lang-Lazdunski L, Hvass U, Pansard Y, Belmatoug N, Faraggi M, et al. Management of giant coronary artery aneurysm with fistulization into the right atrium, *Ann Thorac Surg*. (1993) 56:372-4. doi: 10.1016/0003-4975(93)91182-m
29. Tsujimoto K, Osawa K, Yoshida H, Kuinose M. Giant coronary artery aneurysm in the atrial septum, *BMJ Case Rep*. (2024) 17:e257748 [pii]. doi: 10.1136/bcr-2023-257748
30. Missouris CG, Fluck D, Brecker S. Images in cardiology: Right coronary artery to coronary sinus fistula, *Heart*. (1999) 81:270. doi: 10.1136/hrt.81.3.270
31. Shanmugam G, Bayfield M. Integrated procedure for giant right coronary aneurysm with fistula and atrial fibrillation--coronary grafting, fistula obliteration and radiofrequency maze, *Interact Cardiovasc Thorac Surg*. (2004) 3:168-70. doi: 10.1016/S1569-9293(03)00268-8
32. Upadhyay R, Gargava A, Prabhu V, Sarkar M, Ramteke JH. Coronary cameral fistula and its complications: A case report, *Ann Card Anaesth*. (2020) 23:496-8. doi: 10.4103/aca.ACA_140_19
33. Kawsara A, Núñez Gil IJ, Alqahtani F, Moreland J, Rihal CS, Alkhouli M. Management of Coronary Artery Aneurysms, *JACC Cardiovasc Interv*. (2018) 11:1211-23. doi: 10.1016/j.jcin.2018.02.041
34. Barlis P, Calafiore P, O'Donnell D. Images in cardiology. Angina from a right coronary artery to right atrial fistula, *Heart*. (2006) 92:342. doi: 10.1136/hrt.2005.069872
35. Boulmier D, Bedossa M, Almange C, Le Breton H. Images in cardiology. Large fistula between right coronary artery and right atrium in a 50 year old asymptomatic woman, *Heart*. (2002) 87:454. doi: 10.1136/heart.87.5.454
36. Agatston AS, Chapman E, Hildner FJ, Samet P. Diagnosis of a right coronary artery-right atrial fistula using two-dimensional and Doppler echocardiography, *Am J Cardiol*. (1984) 54:238-9. doi: 10.1016/0002-9149(84)90340-0
37. Kadir I, Ascione R, Linter S, Bryan AJ. Intraoperative localisation and management of coronary artery fistula using transesophageal echocardiography, *Eur J Cardiothorac Surg*. (1999) 16:364-6. doi: 10.1016/s1010-7940(99)00209-2
38. Rognoni A, Ferrero V, Teodori G, Ribichini F. Successful surgical treatment of a giant coronary aneurysm communicating with the right atrium, *J Cardiovasc Med (Hagerstown)*. (2007) 8:1061-4. doi: 10.2459/JCM.0b013e32805f0d09
39. Agrawal DP, Joshi AS, Nm S. The curious case of coronary cameral fistula with infective endocarditis: A rare harbinger of a common clinical entity, *Turk Gogus Kalp Damar Cerrahisi Derg*. (2021) 29:248-51. doi: 10.5606/tgkdc.dergisi.2021.21129
40. Tomai F, Sommariva L, Nudi F, Gioffrè G, Chiariello L. Right coronary artery cirsoid with fistulous connection to the coronary sinus, *Cathet Cardiovasc Diagn*. (1993) 30:310-2. doi: 10.1002/ccd.1810300410
41. Mishra J, Puri HP, Hsiung MC, Misra S, Khairnar P, Laxmi Gollamudi B, et al. Incremental value of live/real time three-dimensional over two-dimensional transesophageal echocardiography in the evaluation of right coronary artery fistula, *Echocardiography*. (2011) 28:805-8. doi: 10.1111/j.1540-8175.2011.01447.x
42. Zoghbi E, Seif F, Obeid M, Abou Nader G, Sawaya J. A young female with an unusual cause of dyspnea, *Int J Cardiol*. (2007) 122:e21-2. doi: 10.1016/j.ijcard.2006.11.068
43. BenAbda R, Gunn A, Roberge E, Yang TW. An unusual cause of atrial fibrillation in a young active duty

soldier, *Radiol Case Rep*. (2017) 12:233-5. doi: 10.1016/j.radcr.2017.01.012

44. Reinders S, Bogaard K, Reichert CL, Knol RJ. A tortuous road for septic pulmonary emboli in left-sided endocarditis, *Can J Cardiol*. (2014) 30:1462.e3-5. doi: 10.1016/j.cjca.2014.06.008

45. Lubarsky L, Jelnin V, Hecht H. Right coronary artery fistula-evaluation by multidector computed tomographic angiography, *Clin Cardiol*. (2009) 32:E6-7. doi: 10.1002/clc.20067
